# Does a service provide safe, effective rehabilitation? An evaluation method for providers and purchasers

**DOI:** 10.1177/02692155241259644

**Published:** 2024-07-25

**Authors:** Derick T Wade

**Affiliations:** Centre for Movement, Occupation and Rehabilitation Sciences (MOReS), Faculty of Health Sciences, 6395 Oxford Brookes University, Headington Campus, Oxford OX3 0BP, UK

**Keywords:** Rehabilitation services, capability, competence, entrustability

## Abstract

**Background:**

Independent organisations monitor the safety and governance of clinical services but do not assess specialist expertise. Peer review can assess service capability but is resource-intense and infeasible.

**The problem:**

How can you ensure a service provides safe, effective rehabilitation? You ask them to provide data as evidence that they can be trusted to do so. This article suggests a structured approach to providing data on entrustability.

**An analogy:**

How is the specialist skill of a doctor in training established? They provide evidence about high-level outcomes (capabilities in practice) related to their speciality. An educational supervisor assesses whether they can be trusted to perform safely and effectively without supervision. The capabilities in practice define their expertise.

**The solution:**

A service can use seven high-level rehabilitation service capabilities, based on the clinical capabilities associated with medical training, with observable indicative descriptors, to collect evidence of their rehabilitation approach. A service must also select four to eight high-level competencies indicating they can rehabilitate their patient caseload safely and effectively. These competencies also need indicative descriptors as evidence of their performance in the service; 11 examples are given.

**Capabilities.:**

The seven rehabilitation capabilities are: using the biopsychosocial model, having a multi-professional team, making a person-centred rehabilitation plan, working collaboratively across all boundaries, tailoring treatments to the patient's needs, ensuring staff have specific competencies required for their caseload, and acknowledging and managing uncertainty and complexity.

**Conclusion.:**

Service providers could use this structured approach to develop and provide users with evidence of their rehabilitation expertise.

## Introduction

How does one define a care home capable of delivering expert rehabilitation? This question emerged when the British Society of Physical and Rehabilitation Medicine (BSPRM) revised its 2013 guidance on specialist care homes delivering rehabilitation.^1^

An answer is vital. Commissioners, professionals, patients, and their families must trust a care home to deliver what it says. Conversely, a care home has no way of assessing, improving, or demonstrating its ability to provide expert rehabilitation because, in the UK, no organisation validates the clinical competence of a rehabilitation service. The existing organisations consider safety, patient satisfaction, and governance. The answer will apply to all rehabilitation services, not just those in care homes.

The answer proposed is a development of the process used to show that doctors training in rehabilitation medicine are capable. Their curriculum gives high-level outcomes, so-called capabilities in practice, described using indicative behaviours, characterising rehabilitation specialists.^2^ Success depends upon the doctor being considered ‘entrustable’; they are trusted to perform safely and effectively. All professions could use the same approach to demonstrate rehabilitation expertise in addition to their professional expertise.^3^

This article suggests rehabilitation services should collect evidence to show they can be trusted to deliver rehabilitation.

### What is a rehabilitation service?

One definition is ‘a rehabilitation service is an accessible, designated inpatient, outpatient or community-based facility where a coordinated, interdisciplinary rehabilitation programme is carried out to optimise each individual client's functional capacity’.^4^

However, there are several service levels, and an international group^5^ suggested three levels of service:

- The **micro** level: the individual patient and their professionals.
- The **meso** level: a group of professionals who collectively manage many patients.
- The **macro** level consists of all meso-services delivering rehabilitation to a population.

This proposal concerns the meso-level, a rehabilitation service in a healthcare organisation.

Rehabilitation has many definitions. The Cochrane Rehabilitation Group's definition^6^ was derived from an extensive review.^7^ The structure and content of effective rehabilitation services have been established empirically.^8^ A general rehabilitation theory has proposed another definition: ‘Rehabilitation aids natural adaptation to the changes associated with illness primarily through a systematic series of catalytic actions, identifying or facilitating changes the person can make’.^9^

Fortunately, all agree that a rehabilitation service delivers:

- a rehabilitation process,
- based on and delivered by a multi-professional team,
- with specialist knowledge and skills,
- working within the biopsychosocial model of illness,
- focused on disability **and**
- working towards goals concerned with social role performance.

### Evaluating rehabilitation services

Peer review of services can assess their expertise^10^ but it is usually based on a retrospective review of medical records, leading to problems.^11^ and its efficacy lacks strong evidence.^12^

The Commission on Accreditation of Rehabilitation Facilities (CARF) reviews rehabilitation services.^13^ It focuses on governance: ‘CARF accreditation as a demonstration of accountability and conformance to internationally accepted standards that promote excellence in your services’. It does not directly assess the rehabilitation quality.

The Care Quality Commission^14^ has had a similar role in the UK: ‘We make sure health and social care services provide people with safe, effective, compassionate, high-quality care, and we encourage care services to improve’. They consider safety, effectiveness, caring and compassion, responsiveness to patients’ needs, and being well-led, but not the ability to meet patients’ clinical needs.

National Health Service (NHS) England's ‘A new community rehabilitation and reablement model’^15^ recommends care home rehabilitation services without suggesting how they are evaluated.

The BSPRM has published 11 documents, shown in Table S1 (Supplemental material), giving standards for many aspects of rehabilitation. One, ‘Specialist Nursing Home Care for People with Complex Neurological Disability: Guidance to Best Practice’, suggested standards in 33 domains, with 52 subsidiary standards.^1^ They were infeasible for routine use .

Last, the Community Rehabilitation Alliance published ‘Community Rehabilitation Best Practice Standards’.^16^ Table S2 (Supplemental material) shows the recommendations and their associated standards. They could be generalised and used, but not easily.

Thus, no publicly available information exists to check that a service provides **rehabilitation**. The data only cover safety and governance arrangements.

### Evaluation of professional performance

A similar difficulty faces organisations responsible for professional performance, such as the General Medical Council and doctors.

A professional is a member of a closed group with ‘a set of values, behaviours and relationships that underpin the trust the public has in the profession’.^17^ Only other professionals can validate professional expertise; initially, this was by an apprenticeship. Later, examinations were used, and from the mid-1990s, curricula for healthcare professions used task competencies to confirm professional status.

In 2005, ten Cate noted a mismatch between task competence and a person's ability to undertake complex professional activities.^18^ ten Cate and Scheele^19^ suggested that entrustable professional activities could bridge the gap between competency and clinical practice.

In 2016, the UK General Medical Council changed the structure of medical curricula so that ‘A key requirement of the revised GMC curricula standards is the development of curricula based on broader, higher-level generic, shared and speciality-specific outcomes of postgraduate speciality training, rather than multiple granular competencies’.^20^

The Joint Royal Colleges of Physicians Training Board implemented this by identifying six **generic** capabilities in practice for all physicians; each speciality added **specialist** capabilities in practice as their high-level training outcomes.^21^ Thirty medical specialities have curricula based on this approach^22^; the rehabilitation medicine curriculum has eight specialist capabilities.^2^

Specialist capabilities in practice constitute a practical definition of a speciality's expertise; they are crucial characteristics that all speciality practitioners should have.

### Ensuring a professional standard – entrustability

The challenge is quantifying professional performance in high-level and complex activities where there is rarely any unambiguous, correct solution.

The approach developed by ten Cate et al.^23,24^ was to use entrustability. Competencies show that a person has performed straightforward tasks safely; entrustability asserts that the person will undertake a complex activity safely in future. As ten Cate^25^ said, ‘Competencies are descriptors of **physicians**, Entrusted Professional Activities are descriptors of **work**’.

The organisation conferring professional status on someone must assess whether the candidate can be trusted to perform at the agreed standard. Knight et al.^26^ explored the relationships between professionalism, trust, and regulation in law and medicine. They question whether evidence and regulation are the only ways to establish professional standards.

Decisions on entrustability are judgments based on evidence collected over time.^27,28^ When assessing individuals, the assessor(s) consider capability (knowledge and skills) and additional attributes such as integrity, dependability, humility (recognising limitations), and agency (being proactive in maintaining standards).^29^ A review gives further information about entrustability.^30^

### The rehabilitation service challenge

**Figure 1.**
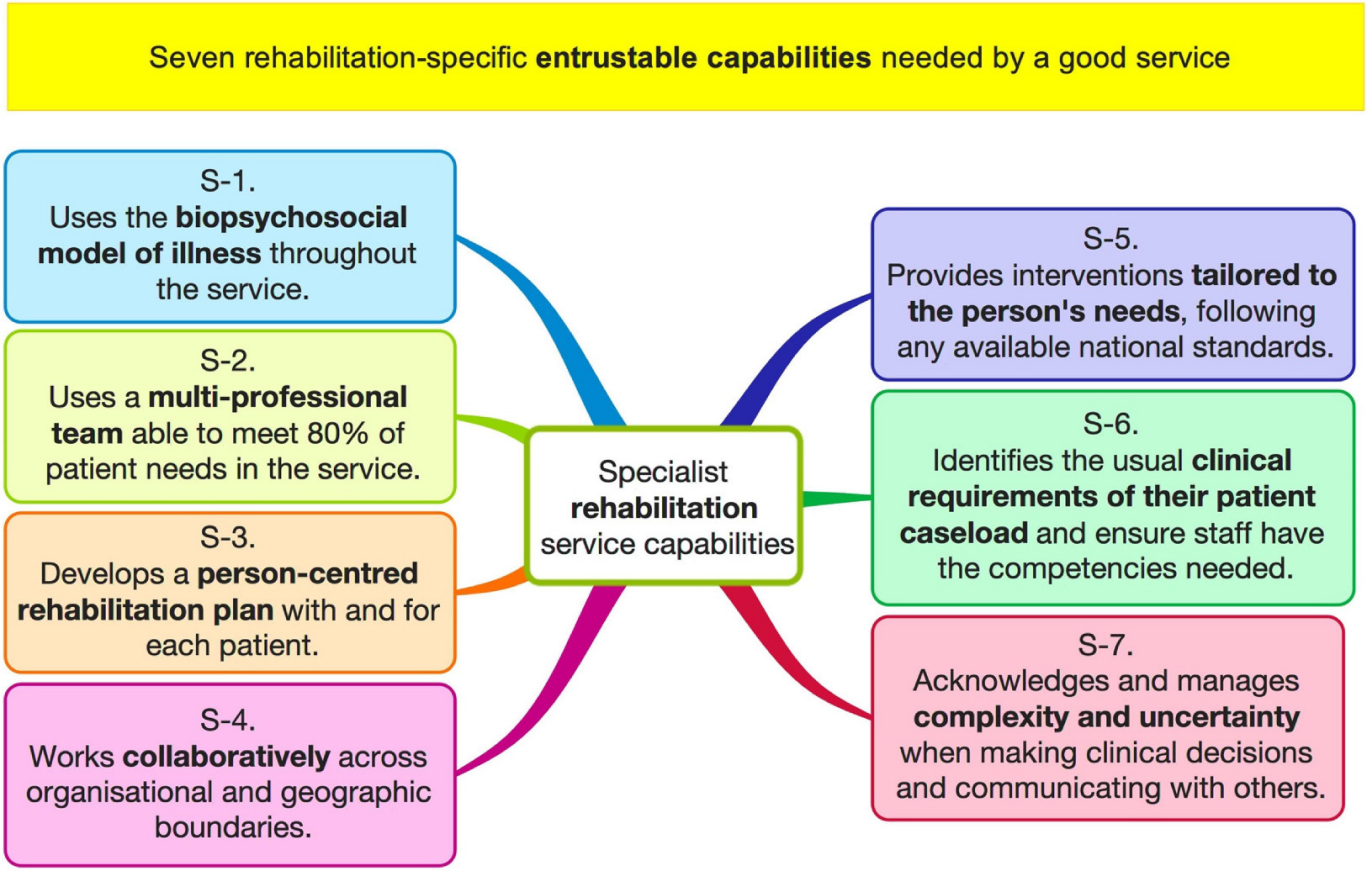
Seven rehabilitation capabilities indicating a service can be trusted to deliver safe, effective rehabilitation.

Rehabilitation is provided by many geographically, managerially, and financially isolated services and typically have different areas of clinical expertise.^31, 32^ The complexity is illustrated in Figure 1 on page 7 of the Community Rehabilitation Best Practice Standards.^16^ Services have different names, such as enablement or intermediate care services. They may be provided in the community, care homes, and hospitals. Much rehabilitation is provided within stroke, geriatric, paediatric, or other NHS services. Independent services, such as care homes, also provide rehabilitation.

No service covers the whole range of rehabilitation, from young children to older adults, from amputees to psychiatric conditions, or from hyper-acute to long-term. Yet they offer rehabilitation. This is like doctors; many provide medical expertise to a multidisciplinary team; some have expertise, but only a few have validated expertise.

Entrustable capabilities covering the service's rehabilitation performance could verify expertise, regardless of caseload, location, or organisation.

### Service capabilities.

The proposed seven **generic capabilities** for non-medical rehabilitation professionals^3^ have been adapted for the new BSPRM nursing home guidance (see Appendix S1, Supplemental material).

The proposed seven **rehabilitation capabilities** (see Figure 1) come from the same source.^3^ They are all prefaced with ‘The rehabilitation service shall …’

1. **Use the biopsychosocial model of illness throughout the service.** The biopsychosocial model of illness^33^ is a defining characteristic of a rehabilitation service.^8^ Everyone, including non-clinicians, should use it in all aspects of their work.
2. **Use a multi-professional team able to meet 80% of patient needs.** A multi-professional team is central to effective rehabilitation.^8^ The core team should be able to manage 80% of clinical problems seen in the service, and there should be procedures for obtaining extra help needed.^34^
3. **Develop a person-centred rehabilitation plan for each patient**. The team must document each patient's formulation and rehabilitation plan; these must be person-centred.^35^
4. **Work collaboratively across organisational and geographic boundaries.** Other services will inevitably be involved with most patients seen, and a rehabilitation service should work flexibly and collaboratively with all other agencies and services in different settings if needed.
5. **Provide rehabilitation interventions tailored to the person's needs.** Effective rehabilitation involves multiple interventions tailored to the patient's needs^8^; the service should not have pre-defined treatment packages. The service should consider published evidence and guidelines when selecting interventions.
6. **Ensure staff have the competencies needed for their patient caseload.** A service must ensure staff have the knowledge and skills to maintain the patient's safety and well-being (meet their care needs) and to provide effective treatment (meet their rehabilitation needs).
7. **Acknowledge and manage uncertainty and complexity.** Every patient's situation is complex, and rehabilitation is a complex process with multiple interventions. Consequently, uncertainty and complexity are hallmarks of rehabilitation. The service must recognise and manage this, for example, using a local peer support network.

### Establishing Entrustability

Table 1 shows some indicative descriptors for each capability. It is vital to appreciate that **indicative** attributes are not comprehensive. They represent key features a service would be expected to have. The service will inevitably have a broader range of attributes, but those given are relatively unambiguous and can be supported by easily collected data.

**Table 1.** The indicative descriptors for the seven entrustable service capabilities. Each capability has a series of observable behaviours or materials (e.g. a policy document) that would be expected in a service delivering rehabilitation. There are many more observable characteristics; this is a selection and is not comprehensive. The absence of a characteristic would not necessarily indicate a lack of capability, but it should increase scrutiny of the capability. The descriptors guide the service and enlarge upon the single sentence defining the capability.

Biopsychosocial model capability
Indicative descriptors
Clinicians use the holistic biopsychosocial model of illness in all areas of clinical practice, for example: to structure all written output such as the formulation, letters, reports, forms to analyse and discuss patient problems within the team to explain a situation to a patient and/or the family, without using jargon Shows familiarity with the concepts and explains its concepts and uses to others The service, including managers, uses the biopsychosocial model when Undertaking quality improvement and audit Planning service development, both internally and externally Teaching rehabilitation to staff in training All training the service offers, internally and externally, incorporates the biopsychosocial model wherever appropriate and relevant.

Multi-professional team use capability
Indicative descriptors
The service employs a range of professionals who can analyse and treat most of the problems seen in the patients. All professionals have specific experience and expertise in rehabilitation in addition to their professional knowledge and skills. For most rehabilitation services, the professions cover medicine, nursing, physiotherapy, occupational therapy, speech and language therapy, and clinical psychology. Other professions may be essential in some services The service has contractual arrangements or agreed protocols to obtain additional professional expertise when needed. There are planned, regular multi-professional team meetings to: Discuss the initial assessment and to formulate a patient's situation. Plan or review a patient's goals and progress. Review and discuss all the patients. Consider service quality, organisation, and development.

Rehabilitation planning capability
Indicative descriptors
For most patients, the clinical documents show that the team: Assessed the patient's losses and abilities, Established the person's interests, priorities, expectations, values, and goals, Met, made a formulation and documented it, Agreed on a plan, which was documented. For most patients, the rehabilitation plan: Was discussed with the patient, Had long-term and shorter-term goals, Was shared with interested parties, Included a planned review process.

Work collaboratively
Indicative descriptors
Documents record the active involvement of professionals from other services, organisations, and settings including: Social Services Community services Hospital services Continuing Healthcare or other commissioners. Formal or informal processes or protocols guide some of the collaborative work undertaken, such as attending regular review meetings with people from other services. The service has links with the local rehabilitation network, if one exists, educational events locally.

Provide rehabilitation interventions
Indicative descriptors
The rehabilitation plans and routine clinical notes show: Reasoning behind some of the decisions to give or not to give an intervention Tailoring of an intervention, such as the influence of a patient's choice or adapting to their need Evidence of review and evaluation of some interventions The service personnel have free, unimpeded access to evidence and guidelines, for example, through the Internet or local library; Team protocols on the management of a particular issue refer to a guideline or other evidence

Relevant competencies and caseload
Indicative descriptors
The service: has a publicly available document summarising the scope of its expertise, which is reviewed annually. Obtains information about a person's needs before admission, confirms that the identified needs can be met safely **or** delays admission until safety can be assured, The service: Has an incident reporting system, Reviews clinical incidents and, when necessary, ensure changes such as training or increased staffing to reduce the risk, Has processes or protocols to access additional predictable clinical expertise when a need is identified, such as a specialist nurse in tissue viability or advice on communication aids. The service: identifies 5–8 key competencies needed as indicators of expertise in rehabilitation tailored to the needs of the expected caseload, reviews the caseload referred and admitted and its range of competencies annually to guide training and recruitment.

Uncertainty and complexity
Indicative descriptors
The service's team members: Have links with other local professionals to discuss specific professional challenges met in their work, Meet regularly as a multi-professional group to discuss challenges in their caseload. The service has links with other local rehabilitation services, which can be contacted informally or formally for advice and support. The organisation responsible for the service proactively organises a team debriefing when a particularly challenging situation has arisen or occurred. Team members have: easy access to resources that might give additional information, such as journals and guidelines: Targeted educational sessions organised for any repeated or frequent situations causing stress.

In medical training, the trainee must collect evidence, primarily about clinical performance across the range of rehabilitation. An Educational Supervisor evaluates this and rates the trainee's entrustability on each capability. When all 14 are entirely entrusted, the trainee receives a certificate.

I propose that the service collects data on the seven specialist capabilities, using the indicators shown in Table 1 as a guide. They could provide other evidence, provided it allows performance evaluation. The service should select information to reassure a commissioner (for example) that they are performing the capability.

Currently, no person or organisation can act as an independent external assessor of a rehabilitation service. Therefore, the potential user, for example, a commissioning clinician, will be the judge (Table S1, Supplemental material).

### Item six – caseload and entrustable competencies

Most services focus on chosen aspects of rehabilitation, and they must demonstrate their ability to manage their patient caseload safely and effectively. This is a crucial parallel with individual professional practice where, after qualification, most professionals develop expertise in a restricted practice area. Professionals must identify and maintain the expertise needed, and rehabilitation services must also do so.

To achieve this, the service must first define its area of interest and then consider what specific knowledge and skills it needs to be safe and effective. In this document, these are referred to as high-level entrustable competencies, and each will need indicative descriptors that act as evidence.

The BSPRM working party for the nursing home guideline has outlined 11 commonly required high-level entrustable competencies. These are shown in Appendix S2 (Supplemental material), which also gives suggested indicators, the likely source of evidence, and refers to relevant guidance.

For example, a service that cares for people with tracheostomies will need to demonstrate the following: ‘The care home provides an environment suitable for people with a tracheostomy including responding to any probable or expected urgent situations, maintaining their safety and wellbeing and monitoring changes in respiratory function’. They will show this by collecting evidence about various indicators (see Appendix S2, Supplemental material).

The crucial feature of this system is that the service must match its competencies to the patient caseload. The service is expected to:

- Define the patient caseload it can accept and manage.
- Select between three and six high-level indicative competencies to cover.
  - The care needed by the patients, and
  - The rehabilitation interventions required.
- Demonstrate that they have the indicative competencies.

The essential feature is that the chosen competencies will represent the many competencies used; if the service demonstrates these, they can be trusted to have the remainder.

The high-level competencies outlined in Appendix S2 (Supplemental material) are unlikely to cover all services. Any care home should consider the indicative high-level competencies needed for their caseload and, if a new one is necessary, develop one based on the general format used here.

### Other evidence

The service can add other evidence to bolster its entrustability.

The dangers of isolated healthcare practitioners are well-known, and professionals should work within a peer group. Thus, an entrustable service should have links to other rehabilitation services, preferably through a rehabilitation network.^16^

Professionals have annual appraisals and plan continuing professional development activities to establish and maintain the expertise needed for their caseload. The service's managing organisation must ensure appropriate team knowledge and skills are available for admitted patients. It must identify when new team knowledge or skills are required and arrange training and staff recruitment. Evidence of an annual caseload and high-level competencies review will increase trust in the service.

## Discussion

This article has taken the approach used by the General Medical Council to ensure the professional speciality expertise of trained doctors in a speciality and generalised it for use when considering specialised services. Figure S2 (Supplemental material) illustrates it, showing four hierarchical levels on the left, a generic method for setting standards for assessing entrustability on the upper right, and its application to a rehabilitation service on the lower right. The technique is fractal-like, repeating from the overall service to individual team members at each level.

Healthcare quality can be assessed using structural, process, or outcome measures.^36^ Governance assessment, for example, by the Care Quality Commission, considers data from all three. The approach used here has focused on structural and process data. Evaluating a rehabilitation service using outcomes would be impossible because:

- Detecting a difference between services is improbable.^37^
- A service would need to be running for several years to give data.
- It only assesses previous service performance.

The principles used in this proposal are now used in training professionals, especially doctors. They have evolved over several decades as a practical method for ensuring suitable expert skills in managing complex situations.

Doubtless, aspects such as the descriptors associated with rehabilitation capabilities can be improved. As the system is used, it should evolve. For example, more specific competencies and better indicative data will be identified.

This proposal can quickly be criticised. For example, there is no evidence of its feasibility. However, I have justified the principles and suggested that processes like this evolve. Moreover, any commissioner can use the method without extra work, which is better than depending on reputation or unstructured opinion. There is no better alternative; widespread regular peer review is not practical.

This system's novel feature is it abolishes the need for a new regulatory body and can be adapted by localities and organisations to suit their needs. This means it is inexpensive, flexible, and likely to change and evolve.

To conclude, the article transfers responsibility for confirming that a service delivers specialist rehabilitation to the service itself, which must provide evidence. I have suggested what evidence will help users trust the service. The system might also assist the service in improving its quality.

**Clinical messages**

- Seven capabilities define rehabilitation expertise for doctors.
- They can be adapted to delineate the rehabilitation expertise of services.
- The system should be feasible.
- It will need to be reviewed and improved.

## Supplemental Material

sj-docx-1-cre-10.1177_02692155241259644 - Supplemental material for Does a service provide safe, effective rehabilitation? An evaluation method for providers and purchasers Supplemental material, sj-docx-1-cre-10.1177_02692155241259644 for Does a service provide safe, effective rehabilitation? An evaluation method for providers and purchasers by Derick T Wade in Clinical Rehabilitation
