# Supplementary material for: Does a service provide safe, effective rehabilitation? An evaluation method for providers and purchasers
Source: Clin Rehabil. 2024 Jul 25;38(9):1147–57. doi: 10.1177/02692155241259644 (PMC11476344; doi:10.1177/02692155241259644)

Does a service provide safe, effective rehabilitation?

An evaluation method for providers and purchasers.

**Supplementary data**

This file has all the information given as supplementary data in the main paper. It uses the titles, such as Table X, from the paper and is in the order they are referred to.

The files are:

- Table one:
  - Rehabilitation guidelines and standards from the British Society of Physical and Rehabilitation Medicine
- Table two:
  - Community Rehabilitation Alliance Recommendations and summary standards.
- Appendix one:
  - Seven generic capabilities applying to rehabilitation services.
- Appendix two:
  - Entrustable competencies for rehabilitation.
- Figure two:
  - An overview of the method for showing a professional person or group providing a clinical activity may be trusted.

**Table one**

Rehabilitation guidelines and standards from the British Society of Physical and Rehabilitation Medicine

| **Title** | **Type** | **Year** |
| --- | --- | --- |
| The role of palliative care interventions for people living with long term neurological conditions | Position paper | 2022 |
| Standards for Specialist Rehabilitation of Spinal Cord Injury. | Standards | 2022 |
| Vocational Rehabilitation: BSRM brief guidance | Guidance | 2021 |
| 2021 BSRM Standards for specialist rehabilitation for community dwelling adults – update of 2002 standards | Standards | 2021 |
| Cancer Rehabilitation BSRM Position Paper | Position paper | 2019 |
| Amputee and Prosthetic Rehabilitation – Standards and Guidelines. 3^rd^ Edition | Guidance & Standards | 2018 |
| Complex regional pain syndrome in adults. UK guidelines for diagnosis, referral and management in primary and secondary care. 2nd edition | National guidance | 2018 |
| Rehabilitation for patients in the acute care pathway following severe disabling illness or injury: BSRM core standards for specialist rehabilitation | Standards | 2014 |
| Specialist Nursing Home Care for People with Complex Neurological Disability: Guidance to Best Practice | Standards | 2013 |
| Long-term neurological conditions: management at the interface between neurology, rehabilitation and palliative care | National Guideline | 2008 |
| Specialised Wheelchair Seating National Clinical Guidelines | National guideline | 2004 |

The guidelines and standards can be accessed at:

<https://www.bsprm.org.uk/resources/>

**Table two**

Community Rehabilitation Alliance

Recommendations and summary standards. [16]

| **Recommendation** | **Service lead’s actions**  **(specifies the standard)** |
| --- | --- |
| Referral processes are explicit, easy, efficient and equitable | Provides a rehabilitation directory, and ensures equality of access and provision of services |
| Rehabilitation interventions are timely, co-ordinated and prevent avoidable disability | Recognises and manages care co-ordination, and delivers/monitors mandatory training |
| Rehabilitation interventions meet patients’ needs and are delivered in appropriate formats | Maps, develops, and describes pathways for patients with different needs |
| Rehabilitation pathways should meet needs and be delivered locally with access to specialist services | Ensures information can be shared and provides resources to be shared with patients |
| Rehabilitation programmes should enable optimisation, self-management, and review | Manages staffing to deliver timely and effective rehabilitation |
| Rehabilitation services are well led, adequately resourced, and linked to other services | Conducts audits and benchmarks services, and identifies service priorities |
| Rehabilitation services involve families | Develops pathways to support families and monitors their experience |

**Reference**

16 Community Rehabilitation Alliance

Community Rehabilitation Best Practice Standards.

<https://www.csp.org.uk/system/files/publication_files/Rehab%20on%20Track_Community%20Standards_ENG_FINAL.pdf>

**Appendix one.**

Seven generic capabilities applying to rehabilitation services.

These standards for **generic clinical activities** are based on those proposed for all non-medical rehabilitation professions. (Wade DT, 2020) They apply to care homes and other organisations providing expert rehabilitation services. They are essential for rehabilitation, just as they are for all other clinical services.

The care home:

1. functions seamlessly within their local healthcare and social support systems.
2. adapts their care and activities to the social context of their residents.
3. communicates effectively with their residents and their families in shared decision-making
4. focuses on quality and safety and participatse in quality improvement systems.
5. understands and support research.
6. teaches and supervises all levels of healthcare staff.
7. bases their clinical practice on the best available evidence and professional standards.

Wade DT.

What attributes should a specialist in rehabilitation have? Seven suggested specialist Capabilities in Practice.

Clinical Rehabilitation. 2020;34(8):995-1003.

<https://doi.org/10.1177/0269215520925869>

**Appendix two.**

Entrustable competencies for rehabilitation.

Many patients transferred to such units for rehabilitation will have complex care needs and will not make progress unless there is expertise to meet these needs.

We have identified high-level competencies that a unit needs to expertly manage common areas of complex care where specialist knowledge and skills are required. As there is currently no mechanism for external, independent checking of capability, we have entitled them as *entrustable* rather than *entrusted*. Moreover, we have termed them *competencies* rather than *capabilities* to distinguish them from rehabilitation capabilities.

We list resources and have developed indicators and that a unit might use to demonstrate that it has such entrustable high-level competencies. These illustrate the principle and cover many of the competencies required to deliver expert complex care.

Other competencies will be needed in some settings. As emphasised in the sixth rehabilitation attribute, each care home service must consider whether it has the appropriate expertise to manage a particular patient. Suppose they are managing a group of residents with problems not covered by these competencies. In that case, they should identify the additional specialist competencies they need so that these can be monitored and developed.

For each identified service competence, we have given:

1. A brief discussion of the patient group and the clinical need.
2. A statement of the competency itself, highlighted in a box.
3. A table showing:
   1. **Observable indicators** on the ***left***
      and, on the ***right*** side:
   2. **Evidence:** likely to be available
   3. **Guidance:** resources to assist service development or understand the expected standards.

These indicators do not comprise a comprehensive list of everything needed, but their presence will show that the service should be safe and effective with the patients seen:

- with a prolonged disorder of consciousness.
- with a communication disorder
- with impaired bowel and bladder excretory function.
- at risk of fixed postural or limb restrictions.
- with difficulties in the oral intake of food and drink.
- needing expert palliative or end-of-life care.
- with or at risk of epilepsy
- with or at risk of skin damage.
- with a tracheostomy.*
- with visual or hearing impairment.
- with severe cognitive and behavioural problems

**Residents with a prolonged disorder of consciousness.**

People with many types of brain damage may develop a prolonged disorder of consciousness (PDOC), such as stroke, Alzheimer’s disease, multiple sclerosis, or brain damage following hypoxia, hypoglycaemia, or trauma. These patients may have other associated difficulties requiring additional expertise.

Service capabilities for these vulnerable residents include the management of the disorder of consciousness, recognition of their loss of mental capacity, and the need to consider the person’s best interests. These residents are severely disabled and frequently have associated difficulties requiring additional clinical expertise.

**The competency**

*Care home staff provide the nursing and medical care required by someone with a prolonged disorder of consciousness. They monitor changes in their level of consciousness to identify deterioration or improvement, support family members, and make decisions in the person’s best interests as required by the Mental Capacity Act 2005.*

| **Indicators** | **Evidence and Resources** |
| --- | --- |
| Staff record **observed behaviours** separate from their **interpretation**. | ***Evidence*** *from notes; may use a form:* <https://doi.org/10.1136/bmjopen-2018-026211> |
| The team minimises the use of drugs and regularly reviews the drugs that are taken. | ***Evidence*** *from team meeting records* |
| The team establishes and records that all decisions and actions are in the person’s best interests. | ***Evidence****: documented best interests processes.* ***Guidance*** *from:*  <https://doi.org/10.1177/0269215519852987> |
| The team pro-actively considers and discusses starting or continuing with life-lengthening treatments | ***Evidence*** *from team meeting records*  ***Guidance from*** <https://www.rcplondon.ac.uk/guidelines-policy/prolonged-disorders-consciousness-following-sudden-onset-brain-injury-national-clinical-guidelines> |
| A treatment escalation plan is discussed with the family and documented soon after admission, and then is reviewed regularly. | ***Evidence*** *from team records.*  ***Guidance*** *from:*  <https://doi.org/10.1177/0269215519852987>  <https://www.resus.org.uk/respect/respect-healthcare-professionals> |
| A palliative care service is easily accessible for symptom control and end-of-life care | ***Evidence****: a protocol, or evidence that one is used* |
| These residents are assessed for associated impairments of vision or hearing that may impact their ability to respond to the environment | ***Evidence****: clinical record* |

**More supporting resources:**

Clinically-assisted nutrition and hydration (CANH) and adults who lack the capacity to consent. Guidance for decision-making in England and Wales.

<https://www.bma.org.uk/media/1161/bma-clinically-assisted-nutrition-hydration-canh-full-guidance.pdf>

Prolonged disorders of consciousness following sudden onset brain injury: national clinical guidelines.

<https://www.rcplondon.ac.uk/guidelines-policy/prolonged-disorders-consciousness-following-sudden-onset-brain-injury-national-clinical-guidelines>

**Residents with a communication disorder**

Communication disorders arise from many different neurological conditions. This may include receptive and expressive aphasia, motor-speech difficulties, dysphonia, and cognitive-communication impairment. The service’s responsibility covers all aspects, including adapting to the environment and using assistive technology.

**The competency**

*Care home staff engage effectively with anyone with communication needs, recognise the person’s impairment, monitor changes in communication, and adapt their communication techniques, including the use of assistive technology.*

| **Indicators** | **Evidence and Resources** |
| --- | --- |
| Training on communication impairment in neurological conditions is provided for staff | ***Evidence:*** *from training records.*  ***Guidance****:*  <https://www.headway.org.uk/about-brain-injury/individuals/effects-of-brain-injury/communication-problems/>  <https://www.stroke.org.uk/what-is-aphasia> |
| The care home has an accessible environment that is aphasia-friendly and meets residents’ communication needs | ***Guidance*** *and standards available from:*  <https://www.stroke.org.uk/what-is-aphasia>  [20162209_InclusiveComms_final.pdf](https://www.rcslt.org/wp-content/uploads/2021/02/20162209_InclusiveComms_final.pdf) |
| A Communication Champion is identified with a specific responsibility to enhance engagement with residents who have communication difficulties | ***Evidence:*** *A specified role; this will be taken on by the Speech and Language Therapist if there is one in the team* |
| All residents with communication difficulties have been assessed by a Speech and Language Therapist. | ***Evidence*** *from clinical records* |
| A communication care plan is in place for all residents with a communication need. | ***Evidence*** *from the care plan which must cover vision, hearing, comprehension, expression, reading, and writing abilities and, if needed, using Augmentative and Alternative Communication (AAC) equipment.* |
| Staff are trained in how to set up and facilitate the use of low- and high-tech communication aids | ***Evidence*** *of links with an Augmentative and Alternative Communication (AAC) service hub, with access to training if needed.* |
| Wi-Fi is available for communication devices to work effectively and to access social media. | ***Evidence*** *of availability to residents.* |
| Residents with impaired dexterity are enabled to access the call bell system and TV/radio with the use of Environmental Control Units if needed. | ***Evidence****: Residents with severe are motor disability have been referred to the regional Assistive Technology Service* |

**Residents with impaired bowel and bladder excretory function.**

Urinary incontinence, faecal incontinence, constipation, and difficulty in the use of toilets is common in care homes. They arise from many diseases, most but not all being neurological. They can be a secondary consequence of immobility, inability to communicate, or anxiety. As incontinence is so common, its causes are often not adequately assessed, and possible rehabilitation or therapeutic approaches are overlooked. There is a close association with sexual dysfunction.

**The competency**

*Care home staff assess residents with bowel and bladder problems, provide appropriate treatment or rehabilitation, and offer respectful personal care and support to people with persistent difficulties.*

*.*

| **Indicators** | **Evidence and Resources** |
| --- | --- |
| Staff use a structured approach to detect, assess, and manage bowel and bladder dysfunction, and seek help from a specialist continence service if they have insufficient expertise | ***Evidence:*** *notes, protocols.* ***Guidance*** *from: Urinary incontinence in neurological disease: assessment and management*  <https://www.nice.org.uk/guidance/cg148>  *Faecal incontinence in adults: management*  <https://www.nice.org.uk/guidance/cg49> |
| Staff use Decision Support Tools for the diagnosis and management of urinary infection | ***Evidence*** *from notes.* ***Guidance*** *from:*   - [Urinary tract infection (catheter-associated): antimicrobial prescribing](https://gbr01.safelinks.protection.outlook.com/?url=https%3A%2F%2Fwww.nice.org.uk%2Fguidance%2Fng113&data=05%7C01%7Cj.burn1%40nhs.net%7C4d60e835202f4cb9f6ff08db351a2e72%7C37c354b285b047f5b22207b48d774ee3%7C0%7C1%7C638162159065518474%7CUnknown%7CTWFpbGZsb3d8eyJWIjoiMC4wLjAwMDAiLCJQIjoiV2luMzIiLCJBTiI6Ik1haWwiLCJXVCI6Mn0%3D%7C3000%7C%7C%7C&sdata=V9%2B2uV6J2xkGLy2o7ZqgV0wK2RO7j0WHD8QH52Lh6rw%3D&reserved=0) - [Urinary tract infection (recurrent): antimicrobial prescribing](https://gbr01.safelinks.protection.outlook.com/?url=https%3A%2F%2Fwww.nice.org.uk%2Fguidance%2Fng112&data=05%7C01%7Cj.burn1%40nhs.net%7C4d60e835202f4cb9f6ff08db351a2e72%7C37c354b285b047f5b22207b48d774ee3%7C0%7C1%7C638162159065518474%7CUnknown%7CTWFpbGZsb3d8eyJWIjoiMC4wLjAwMDAiLCJQIjoiV2luMzIiLCJBTiI6Ik1haWwiLCJXVCI6Mn0%3D%7C3000%7C%7C%7C&sdata=s4R4rvsuE5HrhUdoD8Q6%2F%2FYwh7qOld%2FNzfWV4fKml8s%3D&reserved=0) - [Urinary tract infection (lower): antimicrobial prescribing](https://gbr01.safelinks.protection.outlook.com/?url=https%3A%2F%2Fwww.nice.org.uk%2Fguidance%2Fng109&data=05%7C01%7Cj.burn1%40nhs.net%7C4d60e835202f4cb9f6ff08db351a2e72%7C37c354b285b047f5b22207b48d774ee3%7C0%7C1%7C638162159065518474%7CUnknown%7CTWFpbGZsb3d8eyJWIjoiMC4wLjAwMDAiLCJQIjoiV2luMzIiLCJBTiI6Ik1haWwiLCJXVCI6Mn0%3D%7C3000%7C%7C%7C&sdata=RJA5NNBjEjMpyykUQQ74ktLO%2BbZk%2BPxD7%2FHE3dVrGEI%3D&reserved=0) - [Diagnosis of urinary tract infections: Quick reference tool for primary care for consultation and local adaptation](https://gbr01.safelinks.protection.outlook.com/?url=https%3A%2F%2Fwww.gov.uk%2Fgovernment%2Fpublications%2Furinary-tract-infection-diagnosis&data=05%7C01%7Cj.burn1%40nhs.net%7C4d60e835202f4cb9f6ff08db351a2e72%7C37c354b285b047f5b22207b48d774ee3%7C0%7C1%7C638162159065518474%7CUnknown%7CTWFpbGZsb3d8eyJWIjoiMC4wLjAwMDAiLCJQIjoiV2luMzIiLCJBTiI6Ik1haWwiLCJXVCI6Mn0%3D%7C3000%7C%7C%7C&sdata=sRd8gxtv9wpsa0kFIg96SgiT0anSSSE4%2F928ObIlHio%3D&reserved=0) |
| Staff are trained in and use methods to manage people with neurological bowel dysfunction, including manual evacuation | ***Evidence****: training records/job descriptions.* ***Guidance*** *from:*  <https://www.mascip.co.uk/wp-content/uploads/2015/02/CV653N-Neurogenic-Guidelines-Sept-2012.pdf>  [MTG36, Peristeen Plus transanal irrigation system for managing bowel dysfunction.](https://www.nice.org.uk/guidance/mtg36) |
| Lifelong surveillance ultrasound scanning of residents with spinal paralysis and others at high risk of renal complications and renal or bladder stones | ***Evidence****: clinical records.* ***Guidance*** *from*  <https://www.nice.org.uk/guidance/cg148/evidence/cg148-urinary-incontinence-in-neurological-disease-full-guideline3> (2012)  <https://www.mascip.co.uk/wp-content/uploads/2022/12/SCIST-FINAL-2022.pdf> |
| Staff include goals on the management of bowel or bladder dysfunction in residents’ rehabilitation and care plans. | ***Evidence****: Clinical records.* ***Guidance:***  <https://www.britishjournalofnursing.com/content/professional/the-neurogenic-bladder-developing-a-consensus-bladder-and-bowel-management-pathway-for-people-with-ms> |
| Staff consider sexual function and relationships when assessing a person. | ***Evidence****: demonstrated in the clinical record* |
| Management or rehabilitation plans for people with incontinence specifically includes skin care | ***Evidence:*** *clinical records.* ***Guidance*** *from:*  <https://www.nice.org.uk/guidance/cg179> |

**Residents at risk of fixed postural or limb restrictions.**

People with severe disabling conditions risk developing contractures in their limbs and postural abnormalities affecting their trunk secondary to impaired motor control. These problems occur with most diseases affecting movement, mainly but not only diseases of the nervous system. Some require 24-hour postural management.

**The competency**

*Care home staff assess and review the risk of a person developing irreversible postural or limb deformities, arrange an expert assessment, and provide necessary equipment within a personalised management plan.*

*.*

| **Indicators** | **Evidence and Resources** |
| --- | --- |
| Staff assess all residents on admission for the risk or presence of contractures and postural deformities, recording this using a structured assessment. | ***Evidence****: Record of range of movement and postural assessment; photographs of significant findings.*  ***Guidance:***  <https://www.tandfonline.com/doi/full/10.1080/09638288.2022.2071480> |
| Staff include the prevention or management of joint contractures or postural abnormalities within a 24-hour management plan of residents with identified problems. | ***Evidence****: Splinting and seating plans*  *Splinting charts*  ***Guidance:***  [https://youtu.be/aITUZ63khr0](https://gbr01.safelinks.protection.outlook.com/?url=https%3A%2F%2Fscanmail.trustwave.com%2F%3Fc%3D261%26d%3D6PCY5CpBUZlhLAS3Dq7EF7csxGWyy-FyxK6MIKC4hQ%26u%3Dhttps%253a%252f%252fgbr01.safelinks.protection.outlook.com%252f%253furl%253dhttps%25253A%25252F%25252Fyoutu.be%25252FaITUZ63khr0%2526data%253d05%25257C01%25257CJoel.Dunn1%252540nhs.net%25257C35a2c143267843c9327c08db26144c2e%25257C37c354b285b047f5b22207b48d774ee3%25257C0%25257C0%25257C638145640833911106%25257CUnknown%25257CTWFpbGZsb3d8eyJWIjoiMC4wLjAwMDAiLCJQIjoiV2luMzIiLCJBTiI6Ik1haWwiLCJXVCI6Mn0%25253D%25257C3000%25257C%25257C%25257C%2526sdata%253dvSs2427gjLaNyux7cXZOpSBas8kN%25252BDvAgF93H0noQOo%25253D%2526reserved%253d0&data=05%7C01%7CJoel.Dunn1%40nhs.net%7Cdf6b9cbaa3514e81f77c08db7332328b%7C37c354b285b047f5b22207b48d774ee3%7C0%7C0%7C638230431651357290%7CUnknown%7CTWFpbGZsb3d8eyJWIjoiMC4wLjAwMDAiLCJQIjoiV2luMzIiLCJBTiI6Ik1haWwiLCJXVCI6Mn0%3D%7C3000%7C%7C%7C&sdata=HLCt3CMYuKi9BpMoAP1zxoxO4Qzuu%2B%2F3%2FIIgYv9Fx6s%3D&reserved=0)  [https://vimeo.com/762674497](https://gbr01.safelinks.protection.outlook.com/?url=https%3A%2F%2Fscanmail.trustwave.com%2F%3Fc%3D261%26d%3D9ZWZ5BQoGPVfJF4l554xPQNLqb2pcS80keTlkJsCrQ%26u%3Dhttps%253a%252f%252fgbr01.safelinks.protection.outlook.com%252f%253furl%253dhttps%25253A%25252F%25252Fvimeo.com%25252F762674497%2526data%253d05%25257C01%25257CJoel.Dunn1%252540nhs.net%25257C8eca55b197fb4e9a226708db29faf8c2%25257C37c354b285b047f5b22207b48d774ee3%25257C0%25257C0%25257C638149930133985337%25257CUnknown%25257CTWFpbGZsb3d8eyJWIjoiMC4wLjAwMDAiLCJQIjoiV2luMzIiLCJBTiI6Ik1haWwiLCJXVCI6Mn0%25253D%25257C3000%25257C%25257C%25257C%2526sdata%253dcwZ9SGrzN7Dek2eX9sJychuc1PgGumQFLFx8xbmlH6I%25253D%2526reserved%253d0&data=05%7C01%7CJoel.Dunn1%40nhs.net%7Cb176e564796940f7f56608db2a05343a%7C37c354b285b047f5b22207b48d774ee3%7C0%7C0%7C638149974076371984%7CUnknown%7CTWFpbGZsb3d8eyJWIjoiMC4wLjAwMDAiLCJQIjoiV2luMzIiLCJBTiI6Ik1haWwiLCJXVCI6Mn0%3D%7C3000%7C%7C%7C&sdata=wdR0L17C4V2YuH2DP2wMwplUylEDK0%2FFxOk07tTc%2Foc%3D&reserved=0) |
| Staff stretch limbs and ensure a satisfactory posture in bed, chairs, and wheelchairs. | ***Evidence****: Seating and splinting postures displayed in the room*  ***Guidance:*** <https://www.tandfonline.com/doi/full/10.1080/09638288.2022.2071480> |
| Residents without independent mobility have access to appropriate static seating in their room and a wheelchair to access the home and community. | ***Evidence****: Review of residents' seating provision, particularly those who are nursed largely in bed*  ***Guidance:*** <https://www.who.int/publications/i/item/9789240074521> |
| Staff caring for residents who are immobile, or at risk of or have postural deformities or contracture, receive expert advice and training on posture and contracture management. | ***Evidence****: Records show contact with the wheelchair service or with suitably qualified therapists.*  ***Guidance:*** <https://www.rcot.co.uk/care-homes-and-equipment> |
| Residents are reviewed at an appropriate frequency for the risk or presence of contractures and postural deformities, recording this using a structured assessment. | ***Evidence:*** *record of re-assessments.*  ***Guidance:*** <https://www.tandfonline.com/doi/full/10.1080/09638288.2022.2071480> |

**Residents with difficulties in the oral intake of food and drink.**

Many people in care homes have difficulty eating and drinking, often because of a combination of lacking teeth, an inability to see the food, loss of appetite, difficulty chewing or swallowing, or reduced alertness. Assessing a person’s nutritional state is a fundamental requirement of any healthcare service. Managing more complex problems requires additional expertise from a Speech and Language Therapist and Dietician.

**The competency**

*Care home staff assess residents' oral health, dentition, chewing, and swallowing abilities and monitor their weight and oral intake. They maintain good oral health and ensure the safe intake of sufficient nutrition and fluids, either orally or by using an enteral feeding tube.*

*.*

| **Indicators** | **Evidence and Resources** |
| --- | --- |
| Staff monitor oral health and limit the intake of high sugar foods and fluids. They ensure twice daily brushing of natural teeth with a fluoride toothpaste. | ***Evidence:*** *Commitment to twice daily ‘mouth minutes’ even for residents resisting care.*  *Visits by a dentist, dental therapist or dental nurse.*  ***Guidance:***  <https://www.nice.org.uk/guidance/ng48/chapter/Recommendations#daily-mouth-care> |
| Staff monitor nutritional status and the risk of malnutrition. Dietetic help is available to  1. assess the causes of malnutrition  2. To modify diets and improve nutrition.  3. To support residents receiving enteral feeding. | ***Evidence****: Clinical records. Food and fluid charts. Weight charts and/or other anthropometric measurements. MUST scoring. Visits from a dietician*  ***Guidance*** *from:*   - <https://www.bapen.org.uk/pdfs/must/must_full.pdf> - https://www.nice.org.uk/guidance/cg32 |
| Staff monitor swallowing and the risk of choking. | ***Evidence****: Clinical records.*  ***Guidance*** *from:*   - <https://www.rcslt.org/members/clinical-guidance/eating-and-drinking-with-acknowledged-risks-risk-feeding> - <https://www.rcslt.org/members/delivering-quality-services/managing-risk/> - <https://www.iddsi.org/framework> - <https://www.nhs.uk/common-health-questions/accidents-first-aid-and-treatments/what-should-i-do-if-someone-is-choking/> |
| Residents with swallowing difficulties are assessed by a Speech and Language Therapist in liaison with a dietitian. | ***Evidence****: Clinical records. Care plans. Visits by a Speech and Language Therapist*  ***Guidance*** *from:*   - <https://www.cqc.org.uk/guidance-providers/regulations-enforcement/regulation-14-meeting-nutritional-hydration-needs> - <https://www.england.nhs.uk/commissioning/nut-hyd/10-key-characteristics/> - <https://www.gov.wales/sites/default/files/publications/2019-12/food-and-nutrition-care-homes-older-people-drinking-and-importance-hydration-residents.pdf> |
| Care staff support patients with a swallowing impairment, including modifying their diet and feeding people at risk of aspiration once the risks have been ameliorated and accepted. | ***Evidence****: Clinical records. Care plans.*  ***Guidance*** *from:*   - <https://www.nhs.uk/conditions/swallowing-problems-dysphagia/> - <https://www.iddsi.org/framework> - <https://www.rcslt.org/members/clinical-guidance/dysphagia/dysphagia-guidance/> - <https://www.rcslt.org/members/clinical-guidance/eating-and-drinking-with-acknowledged-risks-risk-feeding/#section-3> |
| Use of the IDDSI framework with a choice of modified diet available | ***Evidence:*** *Clinical records. Care plans.*  ***Guidance*** *from:*   - <https://www.iddsi.org/framework> |

**Residents needing expert palliative or end-of-life care.**

People in care homes are likely to die there, and most care homes have, through experience, developed considerable expertise. Specialist palliative care services with consultants in Palliative Medicine can only be directly involved in a small proportion of residents with palliative needs or who are facing complex, challenging decisions.

**The competency**

*The care home provides palliative care to all those who need it, prepares people for their end-of-life period, and at the appropriate time initiates and carries through a planned end-of-life process*

*.*

| **Indicators** | **Evidence and Resources** |
| --- | --- |
| Undertakes advance (or emergency) care planning for all residents, with regular reviews of the goals of treatment and resident’s priorities, and communicates these to other care providers. | ***Evidence*** *of a ReSPECT plan, local Treatment Escalation Plan (TEP) form, or similar forms used in the locality in clinical notes.*  ***Guidance****:*  <https://www.resus.org.uk/respect> |
| Assesses and holistically reviews residents’ symptoms, setting appropriate plans to control pain or distress. | ***Evidence*** *from clinical records.*  ***Guidance*** *from:*  *E-ELCA modules*: [catalogue](https://portal.e-lfh.org.uk/Catalogue/Index?HierarchyId=0_29&programmeId=29) |
| Identifies the need for specialist palliative care (SPC) referral or advice. | ***Evidence*** *of links with the local palliative care services – such as hospices or Specialist Palliative Care Teams in the community or hospital*  ***Guidance:*** [SPICT – Supportive and Palliative Care Indicators Tool](https://www.spict.org.uk/) |
| Delivers most palliative treatments including subcutaneous infusion of appropriate drugs. | ***Evidence*** *of links with local training in the NHS/Charitable sector.*  ***Guidance:*** NICE Guidance, Care of the Dying Adult.<https://www.nice.org.uk/guidance/ng31>  Overview of topic: [Scottish Palliative Care Guidelines - Syringe Pumps](https://www.palliativecareguidelines.scot.nhs.uk/guidelines/end-of-life-care/syringe-pumps.aspx) |
| Considers and recognises when a patient is probably in their last year of life.  **And then:**  Reviews their treatment goals and medication | ***Evidence*** *of use of validated tools, (e.g. SPICT) and methods (e.g. Gold Standards Framework, commonly used in primary care).* ***Guidance****:*  [Welcome to Gold Standards Framework](https://goldstandardsframework.org.uk/)  [structured-medication-reviews-desdocx-v8docx-2.pdf (icb.nhs.uk)](https://remedy.bnssg.icb.nhs.uk/media/5050/structured-medication-reviews-desdocx-v8docx-2.pdf)  BNSGG guidelines: [structured-medication-reviews-desdocx-v8docx-2.pdf (icb.nhs.uk)](https://remedy.bnssg.icb.nhs.uk/media/5050/structured-medication-reviews-desdocx-v8docx-2.pdf) |
| Assesses mental capacity and able to undertake the best interest process, including discussion on limiting or withdrawing life-sustaining or life-lengthening treatment. | ***Evidence*** *from clinical notes.* ***Guidance*** *from:*  <https://doi.org/10.1177/0269215519853013>  <https://doi.org/10.1177/0269215519852987> |

**Residents with or at risk of epilepsy.**

About 8% of nursing home residents have epilepsy, and the prevalence is higher in patients with brain injury, the population most likely to need rehabilitation. The required expertise has two components:

- the management of drugs to minimise the risk of seizure **and** the risk of harmful adverse effects.
- the management of seizures when they occur.

**The competency**

*The care home identifies people at risk of epileptic seizures, treats acute seizures effectively, and carefully manages their future seizure risk, reducing or withdrawing medication when appropriate.*

| **Indicators** | **Evidence and Resources** |
| --- | --- |
| Staff regularly review the medication of a resident with a history of seizures. | ***Evidence****: Records show involvement of the local epilepsy service in the monitoring, continuation and dosage of antiepileptic drugs, Vitamin D supplementation and the use of emergency medication* |
| Staff actively include the resident and family members in decisions on altering medication. | ***Evidence*** *of patient or family involvement in decisions to start, stop, or change the dosage of drugs taken for epilepsy.* ***Guidance:***  <https://www.england.nhs.uk/personalisedcare/shared-decision-making/> |
| Advance Care Planning to cover likely future urgent or immediate decisions e.g. calling for an ambulance, admission to hospital, missed doses of medication | ***Evidence*** *of advance care planning in the clinical records.* ***Guidance:***  <https://www.england.nhs.uk/publication/universal-principles-for-advance-care-planning/> |
| Staff manage acute generalised seizures safely and write a clear description. | ***Evidence****: Record of seizures and action taken*  ***Guidance:*** <https://epilepsysociety.org.uk/about-epilepsy/first-aid-epileptic-seizures> |
| Staff ensure that the resident’s immediate environment minimises risks during a seizure (cot sides, restraints) | ***Evidence****: protocol giving advice to ward staff.*  ***Guidance:***  <https://www.epilepsy.org.uk/living/safety-equipment/alarms-and-monitors> |
| Staff aware of the difficulties faced by those living with epilepsy and the fear of seizures | ***Evidence:*** *Mature risk management decisions*  *Treatment of associated mood disorder*  *Recognition of non-epileptic events*  ***Guidance:*** <https://www.nice.org.uk/guidance/ng217> |

**Residents with or at risk of skin damage.**

Many care home residents have limited or no mobility. They are at risk of skin damage, which has recently been separated into two types:

- **Pressure ulcers are** “*localised damage to the skin and/or underlying tissue, usually over a bony prominence (or related to a medical or other device), resulting from sustained pressure (including pressure associated with shear). The damage can be present as intact skin or an open ulcer and may be painful*”.
- **Moisture-associated skin damage** (MASD) includes:
- Incontinence-associated dermatitis,
- Intertriginous dermatitis,
- Periwound moisture-associated skin damage,
- Peristomal moisture-associated skin damage.

Skin pressure ulcers cause additional pain and ill health and are generally avoidable; once they are present, every effort must be made to heal them.

**The competency**

*The care home identifies all patients with or at significant risk of skin breakdown, undertakes procedures to minimise risk and heal broken skin, monitors all patients regularly, and complies with any national or local notifications required.*

| **Indicators** | **Evidence and Resources** |
| --- | --- |
| Nurses undertake a pressure ulcer risk assessment within 6 hours of admission, with a full skin assessment if at high risk. | ***Evidence:*** *Procedures and policy documents. Clinical records.*  ***Guidance:*** *NICE quality standards QS89.* <https://www.nice.org.uk/guidance/qs89/chapter/List-of-quality-statements>  <https://cks.nice.org.uk/topics/pressure-ulcers/diagnosis/diagnosis-assessment/> |
| Nurses use an appropriate formal method to detect, assess, record, manage and review areas of skin damage, such as the **aSSKINg** bundle | ***Evidence:*** *Grading and measurement in the clinical record. Pressure Management training.*  ***Guidance*:** <https://www.england.nhs.uk/pressure-ulcers-revised-definition-and-measurement-framework/>  [Pressure ulcer categorisation + ASSKING bundle - YouTube](https://www.youtube.com/watch?v=FkUMYIQDiNw)  <https://www.england.nhs.uk/wp-content/uploads/2021/09/Pressure-ulcer-core-curriculum.pdf> |
| Residents at high risk or who cannot move themselves are regularly repositioned. | ***Evidence:*** *Record of pressure area care and turning*  ***Guidance:*** *NICE QS89 and Clinical Guidance CG179* <https://www.nice.org.uk/guidance/cg179> |
| Residents at high risk or who are unable to move themselves are issued with appropriate equipment and pressure redistribution devices. | ***Evidence:*** *policies, liaison with tissue viability service for equipment, equipment availability, clinical records/*  ***Guidance****: NICE QS89 and CG179* |
| Residents with skin damage receive effective treatment and nutritional support.  Appropriate referral to specialist Tissue Viability Services | ***Evidence:*** *clinical records, policy documents*  ***Guidance:*** [The Role of Nutrition for Pressure Ulcer Management: Advances in Skin & Wound Care (lww.com)](https://journals.lww.com/aswcjournal/fulltext/2015/04000/the_role_of_nutrition_for_pressure_ulcer.7.aspx)  <https://www.nice.org.uk/advice/esmpb2/chapter/Key-points-from-the-evidence> |
| All skin pressure ulcers, including moisture associated skin damage, are reported to the local register and the National Reporting and Learning System | ***Evidence:*** *Policies, ward guidance, incident reporting system, clinical notes.*  ***Guidance****:* <https://www.england.nhs.uk/wp-content/uploads/2021/09/Guidance-for-reporting-pressure-ulcers.pdf> |

**Residents with a tracheostomy.***

More patients are being discharged from the hospital with a tracheostomy, some with an expectation of eventual withdrawal and others anticipating its long-term use. Most go to care homes; some go home. A few also receive ventilatory support.

* *Compiled in consultation with the Respiratory Practice Committee of the Royal Hospital for Neuro-disability*

**The competency**

*The care home provides an environment suitable for people with a tracheostomy including responding to any probable or expected urgent situations, maintaining their safety and wellbeing and monitoring changes in respiratory function.*

*.*

| **Indicators** | **Evidence and Resources** |
| --- | --- |
| All staff in contact with residents who have a tracheostomy can respond to an emergency if one arises. | ***Evidence****: training records, protocols readily available on the unit.*  ***Guidance:***  <https://tracheostomy.org.uk/healthcare-staff/>  <https://tracheostomy.org.uk/storage/files/ITC%20basic%20trachy%20competencies.pdf> |
| Staff follow an individualised tracheostomy care plan that includes a daily tracheostomy care bundle. | ***Evidence****: care plans in clinical records, protocols available to team members.*  ***Guidance:*** Safe Tracheostomy Care – a toolkit for healthcare staff’, Version 2, June 2020 [Safe TrachyCareToolkit V9b.pdf (tracheostomy.org.uk)](https://tracheostomy.org.uk/storage/files/Safe%20TrachyCareToolkit%20V9b.pdf) |
| Standardised bedhead signs which include an emergency tracheostomy algorithm are placed above the bed of all residents with a tracheostomy. | ***Evidence****: signs above the beds or on the wheelchair of all people with a tracheostomy.*  ***Guidance***  [NTSP_GREEN_Tracheostomy_Algorithm.pdf](https://tracheostomy.org.uk/storage/files/NTSP_GREEN_Tracheostomy_Algorithm.pdf)  <https://tracheostomy.org.uk/NTSP-Algorithms-and-Bedheads> |
| Staff can always access emergency equipment. | ***Evidence****: Visible and accessible*  ***Guidance:***<https://tracheostomy.org.uk/healthcare-staff/emergency-care>  [Safe TrachyCareToolkit V9b.pdf (tracheostomy.org.uk)](https://tracheostomy.org.uk/storage/files/Safe%20TrachyCareToolkit%20V9b.pdf)  <https://doi.org/10.1016/j.bja.2020.04.064> |
| Staff keep a personalised tracheostomy document recording the person’s history, key details, and weaning plan | ***Evidence:*** *From clinical records, There is a Tracheostomy Passport available when admitted to hospital*  ***Guidance:***  Example tracheostomy passport <https://www.ccs-sth.org/resources/Documents/Tracheostomy%20Care%20Group/Trachi-Pass%20VERSION%202%20July%2015.pdf> |
| Staff are supported by a specialist professional or tracheostomy team that they can consult by phone, and which visits the unit to review residents | ***Evidence:*** *Protocol for accessing specialist support****.*** *This might be from a local ENT department, an ICU outreach service, or a local tracheostomy support team. There is a Tracheostomy Champion within the unit* |
| The unit shows a commitment to the safety of residents with a tracheostomy | ***Evidence:*** MDT review of adverse incidents and opportunities for clinical governance with other units |

**Residents with visual or hearing impairment.**

Primary impairment of vision or hearing is common, increasing with age. Most people in most care homes will have an impairment of one or both. People with disabilities often do not or cannot indicate that they have problems or are having difficulty using the aids they own. Staff frequently fail to detect visual or hearing [impairment](https://bmcgeriatr.biomedcentral.com/articles/10.1186/s12877-020-01959-0).

**The competency**

*The care home optimises the hearing and seeing of its residents and adapts the environment to the needs of residents with sensory impairments.*

| **Behavioural indicators** | **Comment/resources** |
| --- | --- |
| All residents should be asked if they have difficulty seeing or hearing and whether they use glasses or a hearing aid. | ***Evidence****: Record of asking new residents about their vision and hearing and making some assessment within two days of admission.*  ***Guidance:*** <https://doi.org/10.1136/bmjopen-2018-027803>  <https://www.nice.org.uk/guidance/QS50/chapter/quality-statement-4-recognition-of-sensory-impairment#quality-statement-4-recognition-of-sensory-impairment> |
| Staff ensure all aids are accessible, used, and functioning and this need is prominently recorded on clinical files. Hearing aids are cleaned every day, and the battery is replaced or charged as needed. | ***Evidence****: The presence and management of sensory impairments are included in each care record*  ***Guidance****:* <https://rnid.org.uk/wp-content/uploads/2020/05/A1422_Hear_to_Care_Guide_A4.pdf>  <https://media.rnib.org.uk/documents/Sight_loss_in_older_people_-_Guide_for_GPs.pdf>  <https://www.careengland.org.uk/sensory-loss-care-homes-diagnosis-awareness-response/> |
| The environment is optimised for people with impaired hearing and vision with attention to noise, amplification, illumination, and contrast. | ***Evidence:*** *Record of appropriate equipment provision, environmental adaptation, or referral. A Hearing or Vision Champion*  ***Guidance****:* <https://rnid.org.uk/information-and-support/support-for-health-and-social-care-professionals/guidance-for-residential-care-homes/>  <https://www.rnib.org.uk/living-with-sight-loss/independent-living/practical-adaptations/> |
| The service takes positive actions to improve the lives and reduce the loneliness of people with visual or hearing impairment. | ***Evidence****: Policies, social activities, clinical records.*  ***Guidance****:* <https://www.sscr.nihr.ac.uk/wp-content/uploads/SSCR-research-findings_RF120.pdf>  <https://doi.org/10.1177/0194599820910377> |

**Residents with severe cognitive and behavioural problems**

Care homes are social places, and each resident will interact with other residents and staff in their own way. Usually, their behaviour complies with societal norms but may occasionally or persistently pose a risk to themselves, other residents, staff, or visitors. Such challenging behaviours are seen particularly after brain injury and are closely linked to cognitive impairment. A few care homes specialise in managing this group of patients.

**The competency**

*Care home staff and the home environment keep residents safe whilst promoting the person’s autonomy and well-being with minimal use of physical or pharmacological restraining techniques.*

| **Indicators** | **Comment/resource** |
| --- | --- |
| Staff trained in listening, reassurance, motivation and distraction techniques. | **Evidence:** Formal training programmes, Facilities  **Guidance**:  <https://www.england.nhs.uk/long-read/listening-well-guidance/> |
| Staff assess and monitor behaviour | **Evidence:** Monitoring charts  Measurement scales  **Guidance:** |
| A documented debriefing occurs soon after any major clinical event to reflect and learn from what has happened. Provision of support to front-line staff. | **Evidence:**  Incident reports  **Guidance**: <https://www.nice.org.uk/guidance/ng10>  <https://www.nice.org.uk/guidance/qs154/chapter/quality-statement-5-immediate-post-incident-debrief> |
| A comprehensive and updated formulation is available which explains the resident’s perspective, the meaning of mood and behaviour changes and common precipitants. There is a written plan suggesting appropriate responses to mood or behaviour changes | **Evidence:** Formulations in clinical  record  Consistent response to  target behaviours  **Guidance**  <https://www.challengingbehaviour.org.uk/wp-content/uploads/2021/02/003-Positive-Behaviour-Support-Planning-Part-3.pdf> |
| Senior staff are alerted to expressions of severe distress, hopelessnesss or self-harm/neglect. Access to emergency mental health services. | **Evidence**: Clinical records  Frequency of self harm  **Guidance**: <https://www.nice.org.uk/guidance/ng97>  <https://www.nice.org.uk/guidance/ng225/chapter/Recommendations> |
| Avoidance of sedative medication. Regular review of any medication that is used with a full explanation of why it is justified and its likely benefits and risks. | **Evidence:**Use of major tranquilisers  Use of benzodiazepines  Guidance:  *https://handbook.ggcmedicines.org.uk/guidelines/central-nervous-system/management-of-acutely-disturbed-patients-including-delirium/* |
| Orientation prompts and reminders of rehabilitation and care plans. Family members engaged with staff in the rehabilitation programme | **Evidence**: Written prompts in clinical  areas  **Guidance:**  <https://www.headway.org.uk/about-brain-injury/individuals/effects-of-brain-injury/memory-problems/> |
| Routines of care that promote meaningful activity, exercise and sleep  A calm environment with predictable routines and access to low stimulation areas | **Evidence:** Rehabilitation program  Facilities |
| Appropriate use of the Mental Health Act and Mental Capacity Act | **Evidence:** Assessment of capacity and mental  state. Justification for using  legislation  Regular review of the application  of the MHA and MCA and  imposed restrictions  **Guidance:**  <https://assets.publishing.service.gov.uk/media/62321dcae90e070ed943236f/MCA>  https://www.gov.uk/government/publications/code-of-practice-mental-health-act-1983 |
|  |  |

Some general resources of value

**Active listening.**

- [NHS England » Listening well guidance](https://www.england.nhs.uk/long-read/listening-well-guidance/)

**Motivational interviewing.**

- [Person-centred care | Health Education England (hee.nhs.uk)](https://www.hee.nhs.uk/our-work/person-centred-care)
- [A systematic review of motivational interviewing in physical health care settings - Knight - 2006 - British Journal of Health Psychology - Wiley Online Library](https://bpspsychub.onlinelibrary.wiley.com/doi/10.1348/135910705X52516)
- [Revolutionize Nurse-Patient Relationships: Motivational Interviewing for Nurses - Quenza](https://quenza.com/blog/knowledge-base/motivational-interviewing-for-nurses/#:~:text=Applying%20Motivational%20Interviewing%20Techniques%20in%20Nursing%20Practice%201,Talk%20...%204%20Evoking%20Motivation%20to%20Change%20)

**Recording and monitoring behaviour.**

- [20180705_900824_briefguide-positive_behaviour_support_for_people_with_behaviours_that_challenge_v4.pdf (cqc.org.uk)](https://www.cqc.org.uk/sites/default/files/20180705_900824_briefguide-positive_behaviour_support_for_people_with_behaviours_that_challenge_v4.pdf)
- [Recommendations | Behaviour change: individual approaches | Guidance | NICE](https://www.nice.org.uk/guidance/ph49/chapter/Recommendations)
- [Nick-Alderman-Aggression.pdf (swan.ac.uk)](https://projects.swan.ac.uk/sasnos/wp-content/uploads/2018/12/Nick-Alderman-Aggression.pdf)
- <https://www.nhs.uk/conditions/social-care-and-support-guide/practical-tips-if-you-care-for-someone/how-to-deal-with-challenging-behaviour-in-adults/>
- <https://www.challengingbehaviour.org.uk/wp-content/uploads/2021/02/003-Positive-Behaviour-Support-Planning-Part-3.pdf>

**Incident debriefing.**

- [Quality statement 5: Immediate post-incident debrief | Violent and aggressive behaviours in people with mental health problems | Quality standards | NICE](https://www.nice.org.uk/guidance/qs154/chapter/quality-statement-5-immediate-post-incident-debrief)

**Clinical formulation.**

**Acute and severe patient distress.**

- [Recommendations | Self-harm: assessment, management and preventing recurrence | Guidance | NICE](https://www.nice.org.uk/guidance/ng225/chapter/Recommendations)
- [NHS England » NHS England proposes new mental health access standards](https://www.england.nhs.uk/2021/07/nhs-england-proposes-new-mental-health-access-standards/)
- [Information sharing and suicide prevention: consensus statement - GOV.UK (www.gov.uk)](https://www.gov.uk/government/publications/consensus-statement-for-information-sharing-and-suicide-prevention/information-sharing-and-suicide-prevention-consensus-statement)

**Sedating drugs.**

- [GGC Medicines - Management of Acutely Disturbed Patients, including Delirium](https://handbook.ggcmedicines.org.uk/guidelines/central-nervous-system/management-of-acutely-disturbed-patients-including-delirium/)
- [Management of aggression, agitation and behavioural disturbances in dementia: valproate preparations | Advice | NICE](https://www.nice.org.uk/advice/esuom41)

**Figure two,**

An overview of the method for showing a professional person or group providing a clinical activity may be trusted.


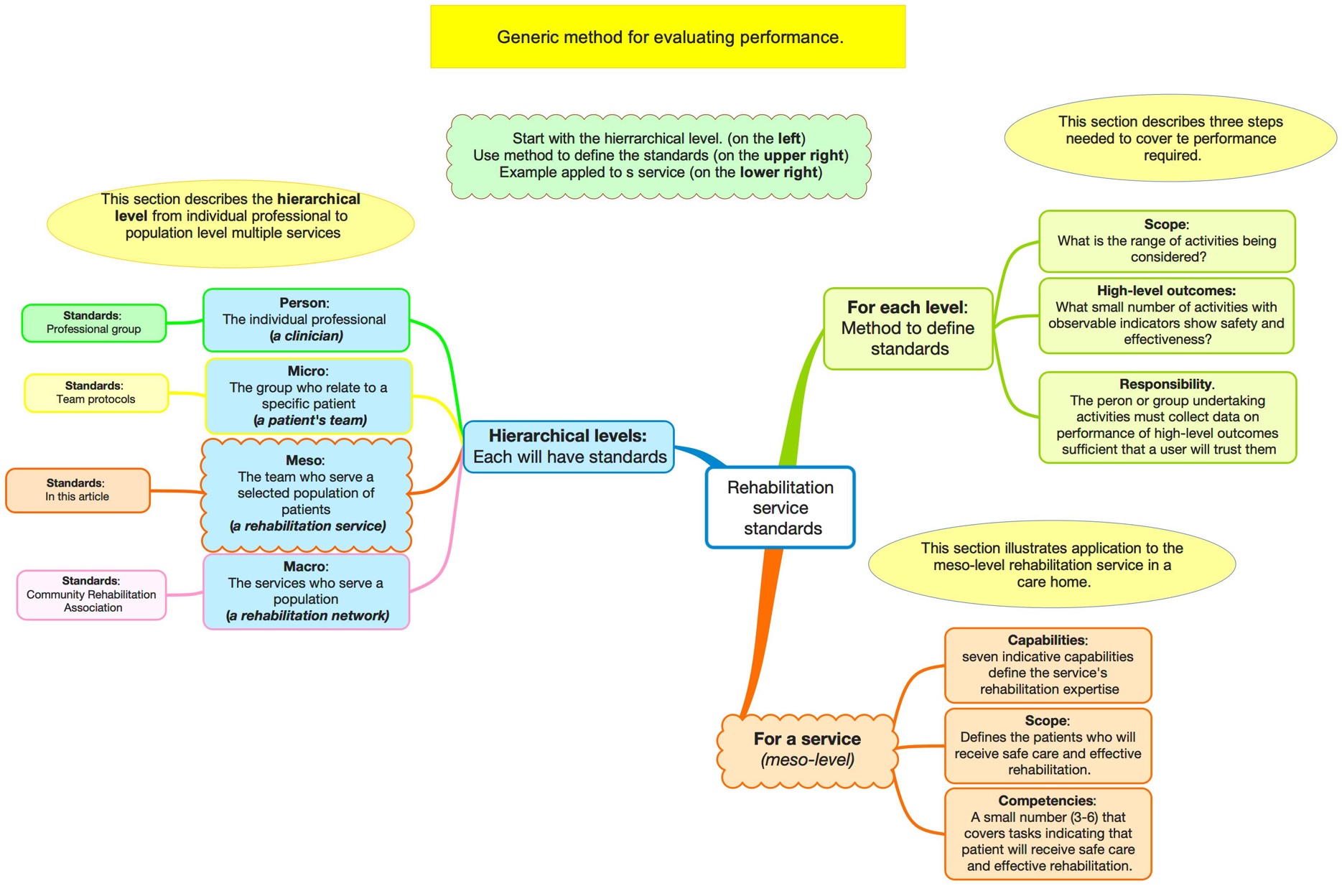

Supplement: sj-docx-1-cre-10.1177_02692155241259644 - Supplemental material for Does a service provide safe, effective rehabilitation? An evaluation method for providers and purchasers [file sj-docx-1-cre-10.1177_02692155241259644.docx]
